# Improved Osteogenesis by Mineralization Combined With Double-Crosslinked Hydrogel Coating for Proliferation and Differentiation of Mesenchymal Stem Cells

**DOI:** 10.3389/fbioe.2021.706423

**Published:** 2021-11-30

**Authors:** Yiqun Ma, Yuwang You, Lu Cao, Bing Liang, Bo Tian, Jian Dong, Hong Lin

**Affiliations:** ^1^ Department of Orthopaedic Surgery, Zhongshan Hospital, Fudan University, Shanghai, China; ^2^ Hospital Infection Management Department, Affiliated Wuxi People’s Hospital of Nanjing Medical University, Wuxi, China; ^3^ Department of Orthopaedic Surgery, Zhongshan Hospital, Fudan University (Xiamen Branch), Xiamen, China

**Keywords:** hydrogel coating, gelatin methacrylate (GelMA), alginate, double crosslink, mesenchymal stem cells

## Abstract

In consideration of improving the interface problems of poly-L-lactic acid (PLLA) that hindered biomedical use, surface coatings have been explored as an appealing strategy in establishing a multi-functional coating for osteogenesis. Though the layer-by-layer (LBL) coating developed, a few studies have applied double-crosslinked hydrogels in this technique. In this research, we established a bilayer coating with double-crosslinked hydrogels [alginate–gelatin methacrylate (GelMA)] containing bone morphogenic protein (BMP)-2 [alginate-GelMA/hydroxyapatite (HA)/BMP-2], which displayed great biocompatibility and osteogenesis. The characterization of the coating showed improved properties and enhanced wettability of the native PLLA. To evaluate the biosafety and inductive ability of osteogenesis, the behavior (viability, adherence, and proliferation) and morphology of human bone mesenchymal stem cells (hBMSCs) on the bilayer coatings were tested by multiple exams. The satisfactory function of osteogenesis was verified in bilayer coatings. We found the best ratios between GelMA and alginate for biological applications. The Alg70-Gel30 and Alg50-Gel50 groups facilitated the osteogenic transformation of hBMSCs. In brief, alginate-GelMA/HA/BMP-2 could increase the hBMSCs’ early transformation of osteoblast lineage and promote the osteogenesis of bone defect, especially the outer hydrogel layer such as Alg70-Gel30 and Alg50-Gel50.

## Introduction

The treatment of bone defects still needs further exploration because of the unsatisfactory clinical outcome. With the increased understanding of tissue engineering and degeneration, polymeric material grafts have been tailored highly biocompatible and widely available so as to reduce rehabilitation time (Rai et al., 2016; Sobolev et al., 2019). Among them, poly-L-lactic acid (PLLA), one of the most applied bioresorbable polymers, opens up a great deal of possibilities in bone substitutes. However, the acidic degradation products released from PLLA may cause an aseptic inflammatory response in bone repair microenvironment and impede bone healing (Kaito et al., 2005). Kandziora et al. (2004) used a fusion cage made of PLLA to treat cervical and lumbar spine diseases. During follow-up, osteolysis occurred around the fusion cage, which is predictive of bone non-union. In order to overcome the shortcoming, several strategies have been designed to modify PLLA, especially the interface between PLLA and adjacent tissues. Based on the potential application in bone engineering, PLLA is usually combined with apatite ceramics, such as hydroxyapatite or tricalcium phosphate, to improve this polymer’s property. The strengthened osteoconductivity and better bone-binding ability provide the conditions for bone regeneration. *In vitro* experiments showed that the hydrophilicity of the PLLA increased and the cell attachment and proliferation enhanced as well (Szustakiewicz et al., 2021; Tan et al., 2021). All this is possible thanks to the component and microstructural similarity between the apatite ceramics and native bone.

Apatite ceramics can also be doped onto the interface of PLLA, which is called biomineralization. It means inorganic crystal, such as hydroxyapatite (HA), is induced to wrap the organic materials. Simulated body fluid (SBF) is widely applied to develop hydroxyapatite coating over PLLA. However, the interface bonding force between HA and PLLA is relatively poor and may last for several weeks. In order to form a negative charge to bind constituents of HA, the surface of PLLA is always pretreated for hydrolysis of carboxyl group. Therefore, polydopamine coating inspired by mussels’ adhesive mechanism has been introduced as the medium to “fix” HA (Lee, Dellatore, Miller, & Messersmith, 2007). The polydopamine coating is tightly bound to the material surface by the exhaustively repeated 3,4-dihydroxy-L-phenylalanine-lysine motif and is easy to administer in alkaline solutions of dopamine. On the other hand, nanoclusters of HA can be secondarily “grown” on polydopamine films (Tsai et al., 2011), and subsequent studies have further increased the generation rate of HA (Tas and Bhaduri, 2004). Chen et al. (2019) used polydopamine (PDA) to biomineralize electrospinning PLLA fibrous membrane, which was excellent for bone tissue repair. A previous study revealed that an organic–inorganic hybrid coating is able to reduce cytotoxicity and improve cell viability, thus prolonging the time of osteogenesis (Li et al., 2016). Hsu et al. (2020) reported that the composition of chlorine-substituted hydroxyapatite/polydopamine on a titanium-64 surface strengthened the osteointegration *in vivo*. Nevertheless, even loose inorganic structures are still unsatisfactory for controlled release of growth factors that induce bone formation.

Except for good biocompatibility and mimicking natural extracellular matrices, hydrogel was found to be the superior carrier for growth factors (Liang et al., 2020). Alginate, a natural polymer, includes (1-4)-linked b-D-mannuronic acid and a-L-guluronic acid with good biocompatibility (Drury et al., 2004; Ahn et al., 2021). With the participation of divalent cations (e.g., Ca^2+^), rapid ionic crosslinking can be achieved in sodium alginate (Ahn et al., 2021). Sodium alginate has been approved by the U.S. Food and Drug Administration (FDA) for medical use, because there is no toxic medium in the sodium alginate gel preparation process (Wang et al., 2020). However, the lack of stem cell adhesion and the uncontrolled degradative kinetics must be addressed (Nair et al., 2020). These defects hampered the proliferation and functionality of the encapsulated cells. Many binding motifs and polymers have been studied, such as matrix metalloproteinase (MMP)-sensitive degradation sequences and collagen, to adjust the compliance with the extracellular matrix (ECM) (Kraehenbuehl et al., 2008; Gregurec et al., 2016). Additionally, a layer-by-layer assembly of chitosan and alginate was applied to modify surface chemistry and expedite the adhesion of human umbilical vein endothelial cells (Silva, Garcia, Reis, Garcia, & Mano, 2017).

Studies have illustrated that modified alginate hydrogels can act as a desired “soil” for the target cells to proliferate and differentiate to the expected phenotypes (Silva et al., 2017; Simo, Fernandez-Fernandez, Vila-Crespo, Ruiperez, & Rodriguez-Nogales, 2017). The gelatin–alginate coating is capable to withstand cyclic compression and even retain proteins entrapped in hydrogel (Pacelli, Basu, Berkland, Wang, & Paul, 2018). Gelatin methacrylate (GelMA), a newly developed photo-crosslinkable hydrogel, has been applied in wound healing and controlled drug release and as a cell culture substrate. For example, Chen et al. (2021) grafted GelMA hydrogel on the surface of expanded nanofiber scaffolds and photo-crosslinked the hydrogel to fit invasive surgery such as laparoscopy and thoracoscopy. Ashwin et al. (2020) made PLLA scaffolds coated with GelMA that were loaded with mucic acid and demonstrated the controlled release of mucic acid and potential capability of osteoblast differentiation. In order to combine gel strength with drug loading performance; achieve better simulation of the biomechanical properties of natural ECM; and improve stem cell adhesion, encapsulation, and differentiation to target phenotypes, a hydrogel coating that is double crosslinked with GelMA and alginate may be a good choice for promoting high-quality bone tissue regeneration.

In consideration of a layer-by-layer (LBL) assembly technique as a successful measurement, it has been explored as an appealing strategy in establishing the multi-functional coating for osteogenesis (Blatchley et al., 2021; Piluso et al., 2021). It could not only regulate local and extended drug release on the surface of PLLA acting as seal barrier but it could also orient the osteogenic differentiation of bone mesenchymal stem cells (BMSCs) and bone formation (Lin, Hsieh, Tseng, & Hsu, 2016; Tsai et al., 2015). Therefore, the aim of this research was to establish a coating to induce osteogenesis of bone mesenchymal stem cells by a combination of alginate and GelMA DN structure for controllable bone morphogenic protein (BMP)-2 release and to check the behavior (adhesion, proliferation, and differentiation) of human bone mesenchymal stem cells (hBMSCs) in the hydrogel platform and its underlying mechanism.

## Materials and Methods

### Materials

PLLA were purchased from Jufukai Biotechnology Co. (Shangdong, China). Sodium alginate, gelatin, dopamine, and Igracure 2959 were purchased from Sigma-Aldrich (St. Louis, MO, United States). Yuanxiang Medical Instrument Co. (Shanghai, China) provided CCK-8 assay kit, alkaline phosphatase (ALP) assay kit, and Dulbecco’s Modified Eagle Medium (DMEM). PCR primers were supplied by Sangon Biotech Co. (Shanghai, China). Total RNA Kit, rhodamine phalloidin, and 4,6-diamidino-2-phenylindole (DAPI) solution were bought from Yeason Biotechnology Co. (Shangdong, China).

### Substrate Preparation and Polydopamine/HA Modification

PLLA composite substrate was synthesized according to our previous report (Cao et al., 2012). Substrate films were dried at −30°C for 24 h after being cast in custom-made Teflon molds. Then, these films were divided into small squares (10 mm in length and 1 mm in thickness) and were stored at room temperature. For biomedical use, the substrate was washed in distilled water ultrasonically and then sterilized with gamma irradiation at 25 kGy.

The prepared substrates were dipped into 2 mg/ ml dopamine alkaline solution in which the pH value was adjusted to 8.5 with 10 mmol Tris-HCl buffer. The solution was stirred carefully to ensure sufficient contact with the PLLA substrate for 24 h. Then, these sheets were washed by distilled water three times to get rid of the free dopamine. The polydopamine-modified PLLA membranes were then dried at 40°C before further use.

Biomineralization was performed largely according to a previous study (Zhang, Zhang, Bao, & Chen, 2014). For rapid synthesis of the HA on polydopamine, first, a simulated body fluid solution, which was used to form HA coating, was prepared according to the work of Tas & Bhaduri (2004) and the pH adjusted to 4.0–4.5 prior to the coating procedure. Second, the polydopamine-modified PLLA membranes were dipped in the above solution with a pH value 6.5 for a certain period; finally, these membranes were denoted as PLLA-PDA-HA, respectively.

### Preparation of Double-Crosslinked Hydrogel Coatings

Sodium alginate was dissolved in phosphate-buffered saline (PBS) at a certain concentration according to Table 1. GelMA was synthesized according to a previous study (Nichol et al., 2010). Next, freeze-dried GelMA and the photoinitiator (Irgacure 2959, Sigma-Aldrich) were dissolved in the prepared sodium alginate solution in a given proportion. The concentration of GelMA solution was set at 0.5%, 1.5%, 2.5%, 3.57, and 4.5% *w*/*v*, respectively. Consequently, BMP-2 (10 μg/ml) was dissolved in the alginate-GelMA solution at 37°C before gel formation. The PLLA-PDA-HA films were dipped into the alginate-GelMA solution for 60 s and withdrawn at a speed of 3,000 rpm for 30 s. The prepared films were dried at room temperature and ionically crosslinked in 100 mM CaCl_2_ solution for 5 min, following irradiation by UV for 15 s with an OmniCure S2000 UV lamp. Finally, the formed double-crosslinked platform was ultrasonically cleaned and sterilized by ultraviolet irradiation for 30 min before cell experiment. According to various component ratios of hydrogel, the samples were denoted as Alg90-Gel10, Alg70-Gel30, Alg50-Gel50, Alg30-Gel70, and Alg10-Gel90 (Table 1). All of them were kept at 4 C overnight.

**TABLE 1:** Labels for functional composition of different ratios and concentration.

Weight ratios (%)		Final concentration (*w*/*v*%)		Labels for composition
Alginate	Gelma	Alginate	Gelma	
90	10	4.5	0.5	Alg90-Gel10
70	30	3.5	1.5	Alg70-Gel30
50	50	2.5	2.5	Alg50-Gel50
30	70	1.5	3.5	Alg30-Gel70
10	90	0.5	4.5	Alg10-Gel90

### Sample Characterization

The morphology of the prepared samples with predetermined LBL coating was observed by field emission scanning electron microscopy (FE-TEM, HT7700, Japan). Atomic force microscopy (AFM, MFP-3D-BIO, Asylum Research, United States) was applied to observe the microstructure and surface roughness. The chemical structures of the double-crosslinked hydrogel were measured by Fourier transform infrared (FTIR) spectroscopy (PerkinElmer Spectrum Two) in the 400–4,000 cm^−1^ range. Energy-dispersive spectrometry was performed by EDS (Oxford Instruments, United Kingdom). Surface hydrophilicity is another factor affecting cell attachment, and it was measured by surface water contact angles by a surface roughness meter (Perthometer M1, Mahr, Germany).

### Cell Culture and BMP-2 Release

hBMSC line was purchased from the Chinese Academy of Sciences (Shanghai, China). The hBMSCs were maintained in culture with Dulbecco’s Modified Eagle’s Medium with low glucose (Low/DMEM) (HyClone, Tauranga, New Zealand) containing 10% fetal bovine serum (FBS, Gibco, United States). It was supplemented with antibiotics (100 U ml^−1^ penicillin and 100 μg ml^−1^ streptomycin) (Gibco BRL) in a humidified incubator with a 5% CO_2_ atmosphere at 37°C. The medium was changed every 2 days. When the cell density exceeded 85%, the cells were digested with trypsin and passed to the cultured cells in a ratio of 1:3.

The cumulative of BMP-2 release was a representative study on the release of growth factor in this double-crosslinked hydrogel (Niu et al., 2009). Five groups of hydrogels (Alg90-Gel10, Alg70-Gel30, Alg50-Gel50, Alg30-Gel70, and Alg10-Gel90) carrying BMP-2 (10 μg per sample) were put in a 2-ml PBS system (pH = 7.4). The solution stayed in a sustained shaking condition at 120 rpm and 37°C for 14 days. At specified time points, 1 ml of solution was sucked out and an equal volume of PBS was added. The measurement of released BMP-2 from each hydrogel samples was carried out by BCA method. The final cumulative release of BMP-2 (%) was calculated as follows: BMP-2 (%) = (total release of BMP2 / total load of BMP2 in the sample) × 100%).

### Cell Viability

Cell adhesion is the first step in facilitating osteogenesis in the scaffold. To check the adhesive effect, the GelMA-alginate hydrogel coating films were put into 24-well plates after sterilization by UV. Then, the hBMSCs were seeded in each hole at a density of 5 × 10^4^/hole for 4 h. Cell viability was determined using Cell Counting Kit-8 (CCK-8) assay. The suspension was transferred into 96-well plates to detect absorbance value.

### Cell Morphology

To verify the morphology of the hBMSCs attached on the coating, the GelMA-alginate hydrogel coating films were co-cultured with hBMSCs for 4 h. The films were gently washed to get rid of impurities and were fixed with 4% paraformaldehyde. Then, the samples were successively stained with 50 mg/ml rhodamine phalloidin for 60 min and with DAPI for 5 min at 4°C. Images were captured by confocal microscopy (Olympus, LX81-ZDC, Japan).

For transmission electron microscopy (TEM) detection, hBMSCs were incubated with GelMA-alginate hydrogel coating films for 4 h at 37°C. Subsequently, after fixing with 4% glutaraldehyde, the fixed samples were dehydrated with a gradient concentration of alcohol (from 30% to 99.5%). The films were dried and sputtered with platinum to test by TEM (HT7700, Hitachi, Japan), respectively.

### Cell Proliferation

The hBMSCs were seeded on different surfaces of the films at a density of 5 × 10^4^ cells/hole in 24-well plates. The samples were cleaned by PBS and stained with live and dead cells for 5 min at 3 days, respectively. The images were captured with a fluorescence microscope. Quantitation of cell proliferation was performed by the CCK-8 test, and optical density (OD) was measured at 450 nm on a microplate reader.

### Real-Time PCR

After seeding hBMSCs for 7 days, the Total RNA Kit was used to extract total RNA of the cells according to the manufacturer’s protocol. Then, the first strand cDNA kits were applied to reverse-transcribe the collected total RNA. Real-time PCR (qPCR) with the Bio-Rad CFX Manager system was performed according to the standard procedure. Amplification was realized by two-step cycling. Melting curves were recorded at a temperature range from 65°C to 95°C. The primers in this study are presented in Table 2.

**TABLE 2:** Primer sequences of osteogenic genes expressed by hBMSCs.

Gene target	Primers sequence (5′-3′)
OCN	Forward primer: 5′ CGTGGCAACTCCATCTT 3′
	Reverse primer: 5′ AGGGTTTCTTGTCCGTGT 3′
BMP-2	Forward primer: 5′ TGA​GGA​TTA​GCA​GGT​CTT​T 3′
	Reverse primer: 5′ TGGATTTGAGGCGTTT 3′
OPN	Forward primer: 5′ TACCTTCTGATTGGGACA 3′
	Reverse primer: 5′ CGAAATTCACGGCTCT 3′
RUNX-2	Forward primer: 5′ GACCACCCAGCCGAACT 3′
	Reverse primer: 5′ CAGCACCGAGCACAGGA 3′

### Alkaline Phosphatase Activity Assay

The hBMSCs were seeded on different surfaces of the films at a density of 5 × 10^4^ cells/hole in 24-well plates until the hBMSCs in each group adhered to the films. Subsequently, the osteogenic medium was prepared consisting of 0.2 mmol/ l ascorbic acid, 100 nmol/ l dexamethasone, and 10 mmol/ l β-glycerol phosphate and applied to the hBMSCs. The medium was changed every 2 days. ALP activity was measured at 4 and 7 days after the use of osteogenic medium by a BCIP/NBT ALP Color Development Kit (Beyotime, China).

### Alizarin Red Staining

The samples were incubated for 28 days with osteogenic medium and fixed with 4% paraformaldehyde for 10 min at 4°C. Alizarin red (pH = 4.2) staining was carried out at 25°C. Images were captured by microscopy.

### Statistical Analysis

Data were recorded as mean ± standard deviation (SD). Student’s *t*-test was used for assessing the difference between two groups, while one-way analysis of variance (ANOVA) was used for evaluating the significance among multiple groups. All the statistical analyses were applied with SPSS 13.0 software (SPSS Inc., United States). Differences were regarded significant if *p* < 0.05.

## Results and Discussion

### Characterization

In our research, polydopamine coating is firstly formed on PLLA films in alkaline and weak oxidation environment, which has an affinity for hydroxyapatite (Wu, Zhang, Zhang, & Chen, 2021). Relevant research found that hydroxyapatite grown on the surface of dopamine in simulated body fluid is more uniform and more consistent with the extracellular environment of bone tissues than hydroxyapatite mixed in hydrogel (Li et al., 2016; Z.; Wang, Chen, Wang, Chen, & Zhang, 2016). Meanwhile, the outer-layer alginate/GelMA pairs provide enough strength to contain growth factors. The design has obvious advantages: firstly, the microstructure of hydroxyapatite is helpful for achieving the three-dimension-stimulating bone environment for osteogenic-associated cells to grow in. Secondly, the covering alginate/GelMA DN, acting as a barrier, would be beneficial for sustained release of BMP-2, resulting in extending its biofunction toward hBMSCs in a relatively long term (Hu et al., 2012).

**FIGURE 1:**
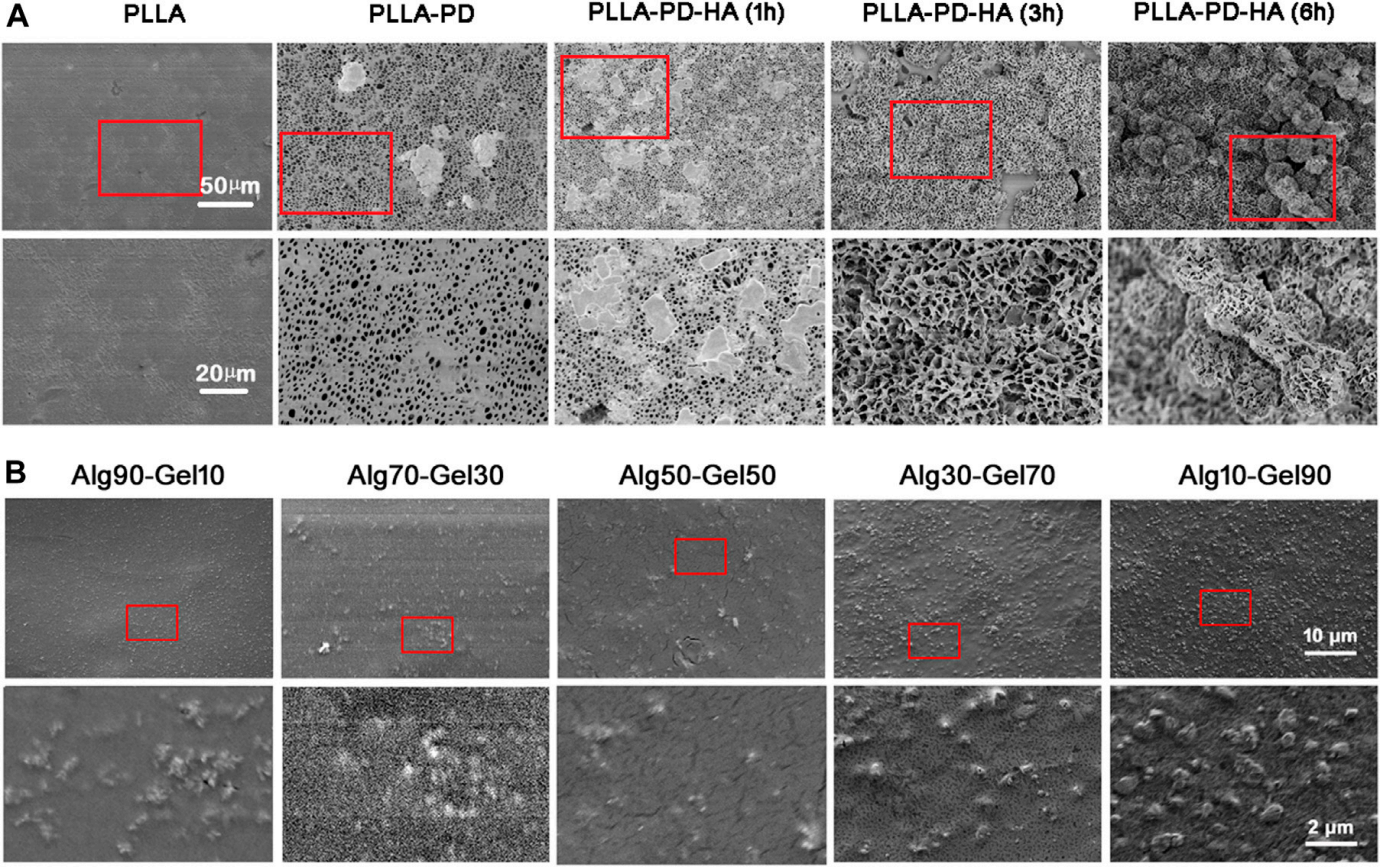
SEM images of **(A)** synthesis of composite coatings: PLLA, PLLA-PD, PLLA-PD-HA (1 h), PLLA-PD-HA (3 h), and PLLA-PD-HA (6 h) and **(B)** different ratios of alginate and GelMA. SEM, scanning electron microscopy; PLLA, poly-L-lactic acid; PD, polydopamine; HA, hydroxyapatite; GelMA, gelatin methacrylate.

Scanning electron microscopy (SEM) and AFM were used to observe the surface morphology of PLLA substrates. As shown in Figure 1A, the native PLLA displayed a relatively flat surface feature. The synthesis of polydopamine on the PLLA surface formed an eruptive microporous structure. With the assistance of strengthened simulated body fluid, well-aligned and highly ordered HA clusters were gradually constructed onto the PLLA films. The piles of HA cluster increased with the prolonged time of simulated body fluid on the PLLA. The pore diameter of the HA was approximately 1–5 μm and was distributed at regular intervals. After covering the alginate/GelMA DN outer layer in different proportions, the sample surfaces were scattered with various degrees of projections (Figure 1B). As sodium alginate is more viscous than GelMA at the same concentration, the protuberance on substrate surface becomes more obvious as the proportion of sodium alginate in the outer coating decreases.

**FIGURE 2:**
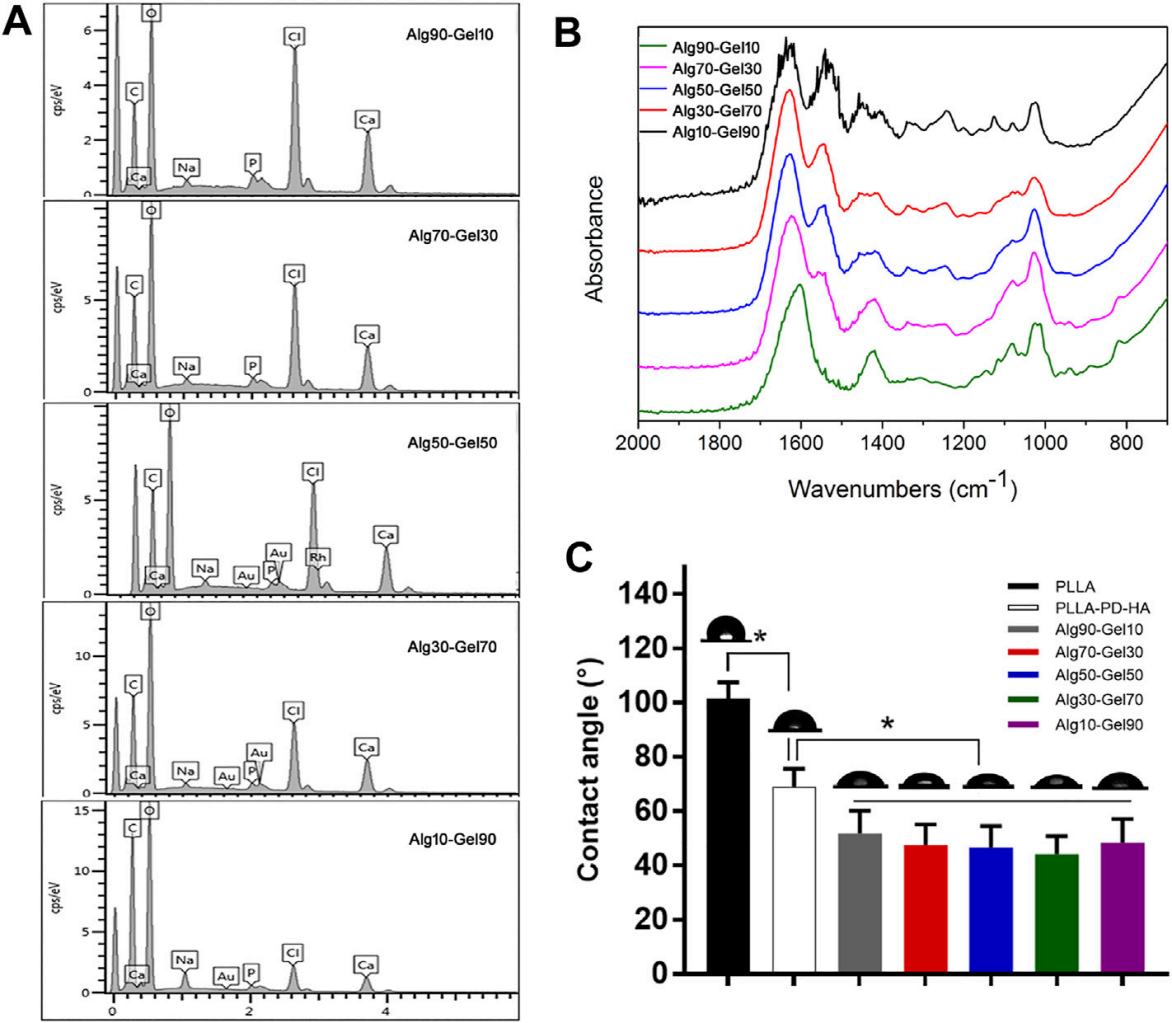
**(A)** SEM-EDS and **(B)** FTIR spectra for Alg90-Gel10, Alg70-Gel30, Alg50-Gel50, Alg30-Gel70, and Alg10-Gel90. **(C)** Contact angle measurement of water on the surface of different samples. FTIR, Fourier transform infrared.

To measure the distribution of element of the coatings, SEM-EDS was performed for hydrogel analysis. As presented in Figure 2A, the hydrogel coatings displayed largely characteristic C, O, H, and Ca elements. The Ca element was derived from hydroxyapatite. In each group, the amount of Ca was presented ∼3%. Meanwhile, with the increase of GelMA in the outer layer, the peak of C and O element content gradually accumulated, attributing to the substitution of the methacrylic anhydride for the amino groups of GelMA molecules (Table 3). The result of SEM-EDS indirectly manifested the successful coverage of multilayer coating on the PLLA films.

**TABLE 3:** Distribution of elements in composition of different ratios (%)

Elements	Alg90-Gel10	Alg70-Gel30	Alg50-Gel50	Alg30-Gel70	Alg10-Gel90
O	40.87	46.97	45.55	53.82	65.99
Na	0.71	0.75	0.73	0.70	4.26
P	1.88	1.57	0.99	1.37	1.51
Cl	29.99	27.30	27.08	22.52	13.25
Ca	26.54	23.42	22.44	21.59	14.99

To further verify the double-crosslinked hydrogel, FTIR spectra (Figure 2B) showed a similar trend in the hybrid hydrogel composed of GelMA and alginate. The characteristic band peaks of the alginate range of 1,460–1,649 cm^−1^ correspond to carboxylate ions and those of 935–1,107 cm^−1^ relate to C–O of the pyranose ring (Golafshan, Kharaziha, & Fathi, 2017; Craciun, Barhalescu, Agop, Ochiuz, & Medicine, 2019). In addition, the spectrum of GelMA consisted of the peak of C=O at 1,630, 1,548, and 1,240 cm^−1^. The C–N–H at 1,500–1,570 cm^−1^ could also be recognized. These characters illustrated the reaction of anhydride methacrylate with free amine groups of gelatin (Rahali et al., 2017). The spectrum of Alg90-Gel10, Alg70-Gel30, Alg50-Gel50, Alg30-Gel70, and Alg10-Gel90 presents broad band peaks at 1,621 and 1,557 cm^−1^ probably because of overlapping with the functional groups of the GelMA and alginate composition, implying the successful introduction of the double-crosslinked hydrogel on the surface of PLLA sheets. Moreover, it semi-quantitatively analyzes specific reference bands of functional groups according to the two components of the coatings, which were less affected by the chemical reaction.

Hydrophilicity is another necessary requirement for the osteoblast-related cells to access bone tissue implants (Oliveira et al., 2021). Figure 2C displays the water contact angles on the surfaces of the substrate and customized coatings, of which, native PLLA showed a contact angle of 101.40°, while mineralization showed an obviously hydrophilic contact angle of 68.12°. After incorporation of hydrogel, there was a further decrease of contact angle to 51.86°–44.24°. In addition, various proportions of hydrogel components did not affect the change of the contact angle. The result may reflect the corresponding deposition of alginate-GelMA/HA bilayers. The hierarchical microstructure may promote hydrophilicity in a progressive behavior, which has a capillary function to facilitate the drop spreading and coincides with a previous report (Xing et al., 2020). These results demonstrate the successful construction of alginate-GelMA/HA bilayer coating on the PLLA films.

### Cell Viability, Attachment, and Proliferation

**FIGURE 3:**
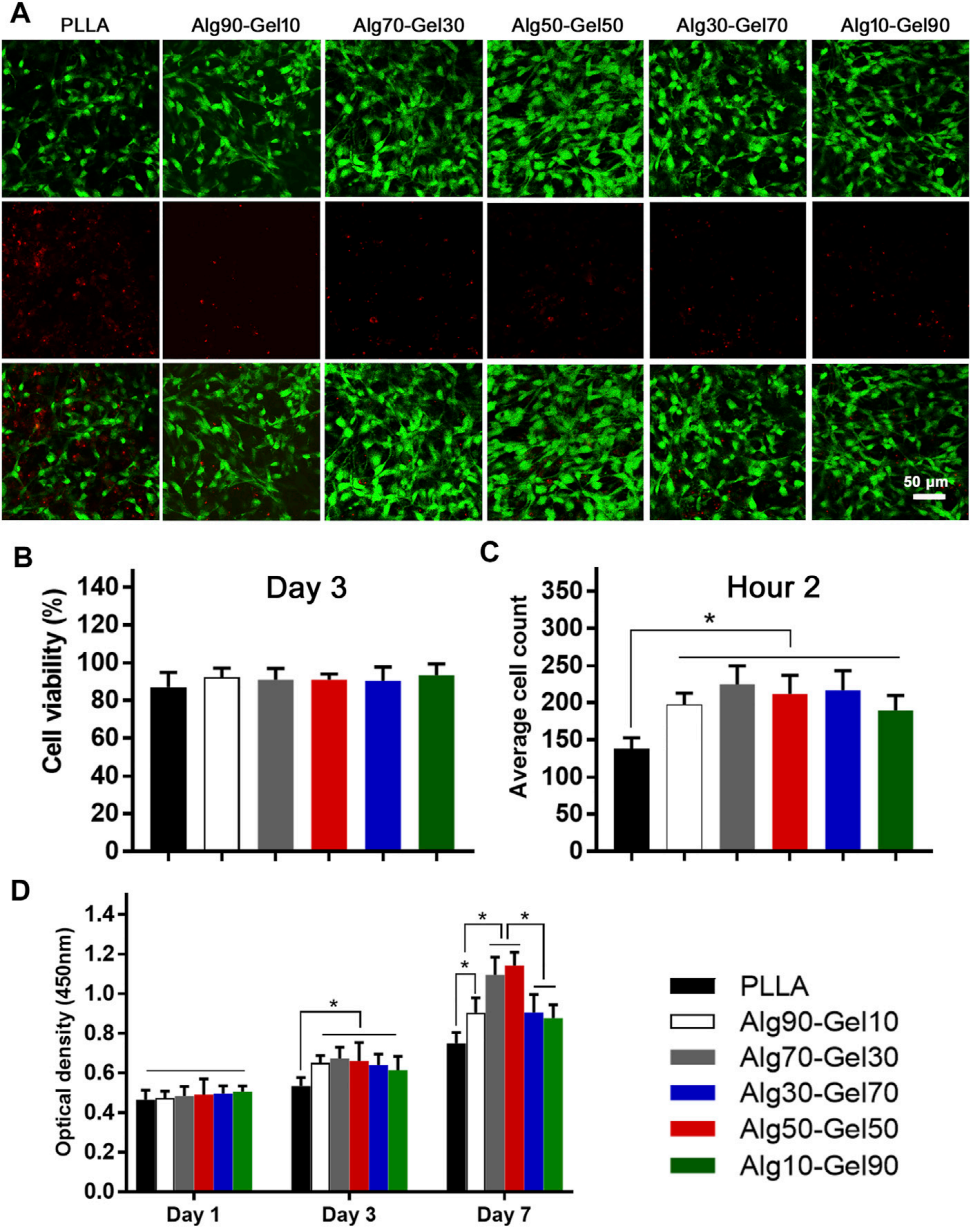
**(A)** Cell viability of human bone mesenchymal stem cells (hBMSCs) on the different samples (scale: 50 μm) and **(B)** statistical analysis. **(C)** The short-term cell adhesion at hour 2 and **(D)** cell proliferation at days 1, 3, and 7 on the PLLA and bilayer coatings (*p < 0.05 compared with the control).

Cell adhesion, growth, and differentiation are dependent on the degree of biocompatibility. To evaluate the cytotoxicity of the coated samples, the survival rate of cells on the coating surface of each group was determined at the same magnification by staining with dead cells on the third day (green dot: live cells; red dot: dead cells) (Figure 3A). Cell viability was assessed by fluorescence microscopy. Although the proportion of dead cells increased slightly in the PLLA group, there were no significant dead cells in each group, indicating that the bilayer coatings in each group had good biosafety and provided an appropriate growth environment for hBMSCs (Figure 3B). It is remarkable, however, that there were fewer viable cells in the native PLLA films than in the coatings, which may result in short-term cell adhesion and relative long-term cell proliferation.

After seeding the cells on the samples for 2 h, quantification was performed to measure the cell attachment according to a previous study (Zhang et al., 2018) (Figure 3C). Notably, the bilayer coatings facilitated the cells adhering on the substrate rather than the bare PLLA film. The OD values of CCK-8 had nearly identical results in the Alg90-Gel10, Alg70-Gel30, Alg50-Gel50, Alg30-Gel70, and Alg10-Gel90 groups, but were higher than those in the PLLA group. To further evaluate the proliferation of cells in each samples, the number of live hBMSCs was measured by CCK-8 test on days 1, 3, and 7 (Figure 3D). Cell numbers increased in all groups, indicating that not only all the bilayer coatings but also the native PLLA provided a suitable microenvironment for cell growth. There were no differences in hBMSCs among the groups on the first day. The proliferative rate in the bilayer groups began to present higher than that in the PLLA alone on day 3. Finally, on day 7, the cell proliferation in the Alg70-Gel30 and Alg50-Gel50 groups did an outstanding performance than that in the other five groups, among which the cell growth of the Alg90-Gel10, Alg30-Gel70, and Alg10-Gel90 groups was far superior to that of the PLLA group. These results illustrated that the double-crosslinked hydrogel combined with HA has the advantage to attract osteoblast-related cells more rapidly to adhere to the surface of the substrate.

### Cell Morphology

**FIGURE 4:**
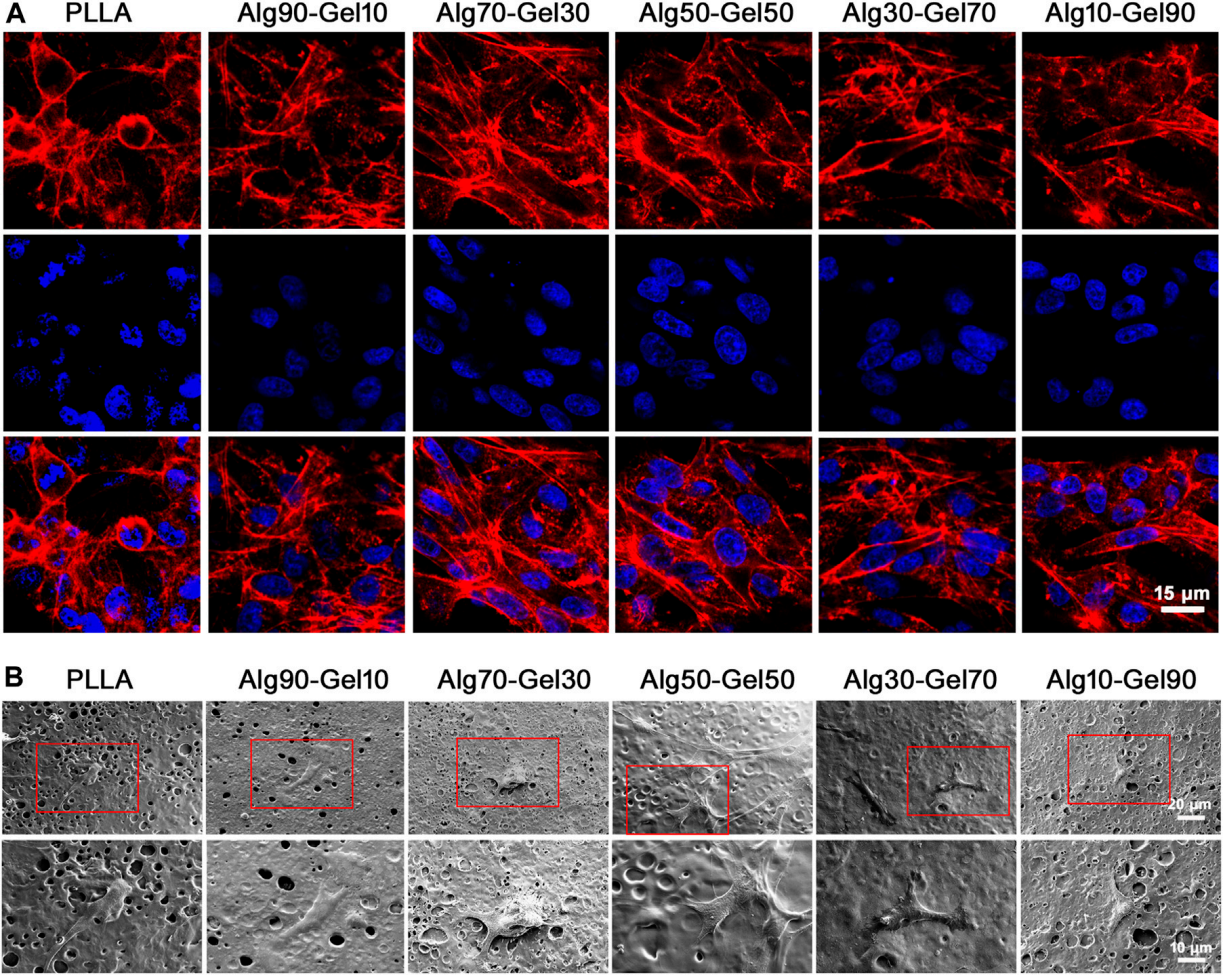
**(A)** Confocal microscopy images of hBMSCs on different groups at 2 h (red, rhodamine phalloidin; blue, DAPI for nucleus) (scale: 15 μm). **(B)** SEM images of hBMSCs in different groups at 3 days.

Additionally, the function of hBMSCs corresponds with a well-ordered morphology. Immunofluorescence detection at hour two showed the intracellular microstructure on the surface of the PLLA sheet and bilayer coatings in different proportions. The cytoskeleton was stained with rhodamine-labeled phalloidin, and nuclei were stained with DAPI by confocal microscopy (Figure 4A). Most of the hBMSCs that adhered to the PLLA film seemed spherical, while the cells on the bilayer coatings had many obvious cytoskeleton/microfilaments, as well as clear intercellular connections. Particularly, a better directional rearrangement of the cell cytoskeleton was displayed in the Alg70-Gel30 and Alg50-Gel50 groups.

To avoid the interference of fluorescence on cell microstructure, SEM was applied to observe the activity of cells on the coating after 3 days of seeding on the samples (Figure 4B). At higher magnification, there were a few hBMSCs on the surface of the native PLLA, and their morphology tended to be elongated. Intercellular connections between cells were not detected. Cells in the Alg90-Gel10 and Alg10-Gel90 groups began to develop a typical fusiform shape and clearly extend their pseudopods, but the cell size was smaller and the intercellular filaments were still not as clear. In the Alg70-Gel30, Alg50-Gel50, and Alg30-Gel70 groups, we found a longer irregular fusiform structure; the pseudopods were normal size, and the number of cells on the surface of the material increased significantly. For the Alg70-Gel30 and Alg50-Gel50 groups, the extended microstructure with some cell–cell interactions was easy to observe, and the pseudopods tended to extend to the microspores in multiple directions. These results showed that the adhesion and migration of hBMSCs in the Alg70-Gel30 and Alg50-Gel50 groups were significantly better than those in other double-crosslinked coatings, especially in the PLLA group.

### Sustained Release of BMP-2 and Expression of Gene

**FIGURE 5:**
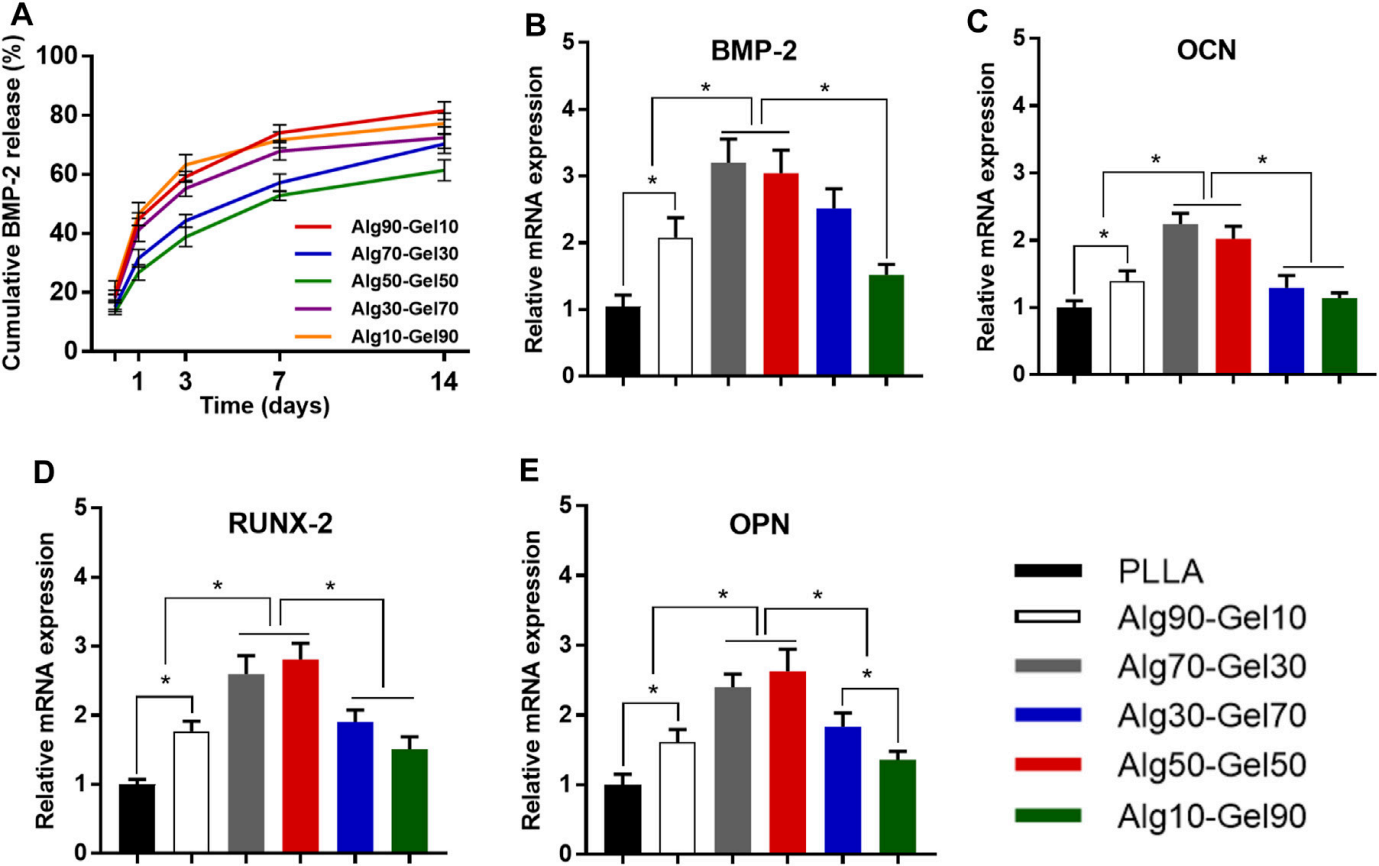
**(A)** Release curves of BMP-2 from different double-crosslinked hydrogels. The mRNA levels of **(B)** BMP-2, **(C)** OCN, **(D)** RUNX-2, and **(E)** OPN were quantified from hBMSCs cultured in various substrates (*p < 0.05 compared with the control).

Figure 5A shows an *in vitro* BMP-2 release curve from different ratios of double-crosslinked hydrogels. All the hydrogel systems displayed a sustained release for 2 weeks. In the first 2 days, the release curve of each group showed a relatively steep but not a burst release. The next 5 days, all the groups exhibited a “plateau” release curves and then entered a process of steady and moderate release. The total BMP-2 release of Alg90-Gel10, Alg70-Gel30, Alg50-Gel50, Alg30-Gel70, and Alg10-Gel90 reached 74.08%, 57.12%, 52.84%, 67.81%, and 71.64%, respectively. Then, the additional release of BMP-2 was less than 10%. Since the BMP-2 was kept inside the hydrogel, it is helpful for hBMSCs and osteoblasts attached or grown into the coatings to proliferate, differentiate, and induce bone formation. Moreover, the results seemed to show the higher crosslinking hydrogel as a barrier against growth factor release. On 2 weeks, the BMP-2 loading capacity of five samples was 18.44%, 29.65%, 38.64%, 27.55%, and 22.70%, respectively, and the drug encapsulation efficiency of Alg50-Gel50 hydrogels was 20.20% and 16.94% higher than that of the Alg90-Gel10 and Alg10-Gel90 groups.

The differentiation of hBMSCs toward osteoblast lineage could be valued by specific osteoblast mRNA expression. The expression of the four osteogenic-related genes (RUNX-2, BMP-2, OCN, and OPN) was detected by RT-PCR experiments on day 14 (Table 3). The cells cultured on PLLA films in DMEM were set as negative control for each experiment, which failed to extract osteogenic-specific genes. In the positive control group, an osteogenic medium, which replaced DMEM, was used to culture hBMSCs on PLLA films. We detected osteogenic-specific gene expression in the positive control group that we treated as reference for other groups. By the 14th day, the expression levels of RUNX-2 and BMP-2, which are the markers of early osteoblast differentiation and bone formation, was higher in the Alg70-Gel30 and Alg50-Gel50 groups than in other bilayer coatings. Meanwhile, the expression of OCN and OPN, which represents late osteogenic formation, showed a similar trend (Figure 5).

### Osteogenic Differentiation

**FIGURE 6:**
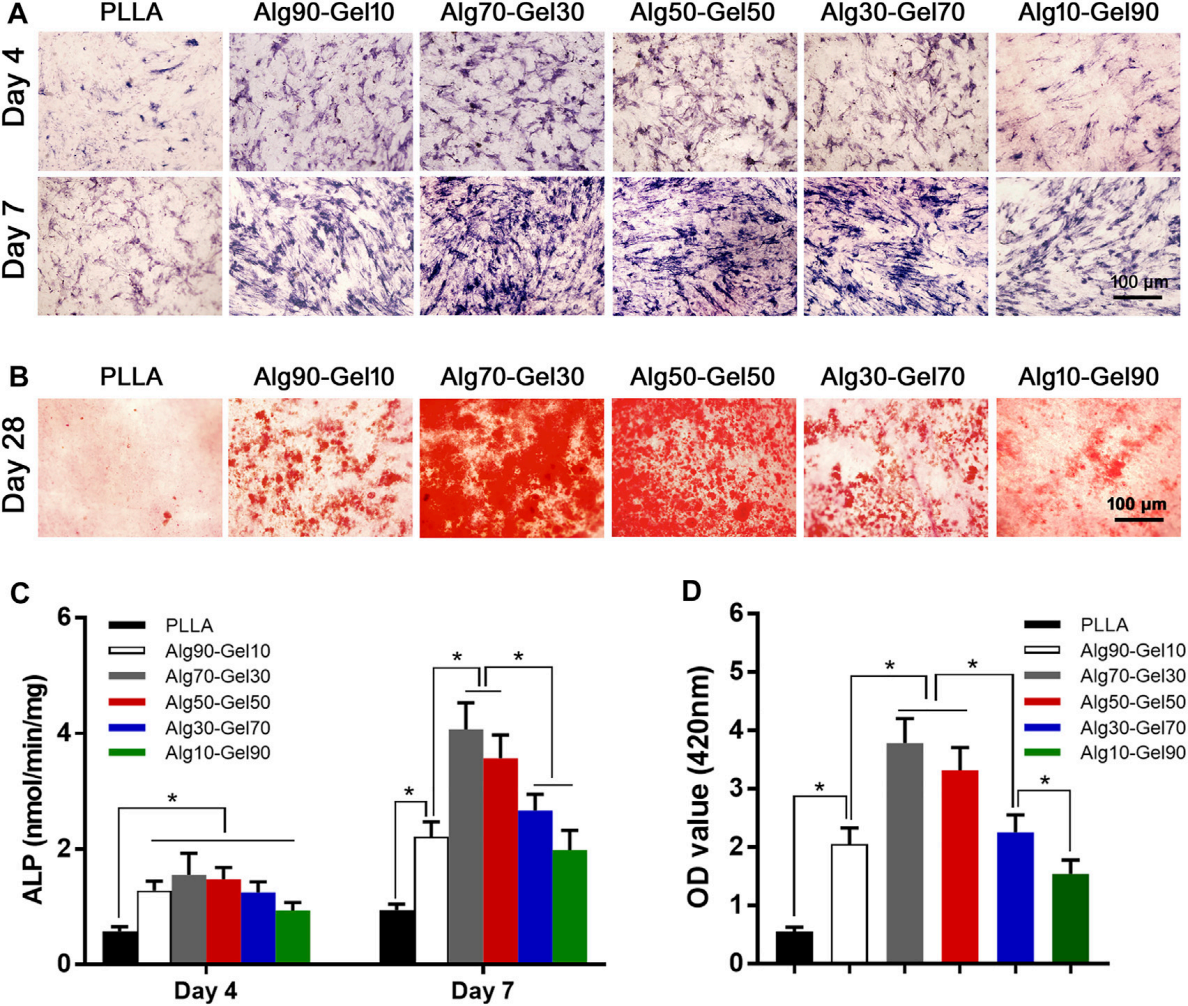
**(A)** ALP staining at 4 and 7 days (scale: 100 μm). **(B)** Alizarin red staining images at 28 days (scale: 100 μm). Quantitative results of **(C)** ALP activity and **(D)** Alizarin red staining (*p < 0.05). ALP, alkaline phosphatase.

To reveal the sustained promotion of osteogenesis of hBMSCs on the coatings, alkaline phosphatase staining was applied to detect ALP expression. After hBMSCs were seeded on the surface of different double-crosslinked coatings and constantly induced with osteogenic medium, the staining was carried out on day 4 and day 7 (Figure 6A). From the perspective of density, the samples in the Alg70-Gel30 and Alg50-Gel50 groups are more deeply stained compared with the other coating groups and the PLLA group. It indicates that early-stage differentiation could occur in Alg70-Gel30 and Alg50-Gel50 bilayer coatings. To accurately depict the early osteogenesis in these groups, the ALP activity was used in quantification. The ALP expression activity in each group enhanced as the time went on. Among them, the highest was in the Alg70-Gel30 and Alg50-Gel50 groups, which coincided with the result of staining (Figure 6C).

At the end stage of osteoblast differentiation, the formation of mineralized nodules is a crucial phenomenon that indicates the maturation of osteoblasts. To evaluate this, Alizarin red staining was used to quantify the degree of mineralization by measuring the number of calcium nodules (stained red spots). Alizarin red staining was conducted on day 28 (Figure 6B). The Alg70-Gel30 and Alg50-Gel50 groups had significantly more nodules compared to every other groups (Figure 6D). The poor performance of mineral nodules in the PLLA group may result from the lack of BMP-2 release “barrier layer.” In consideration of the controlled release of BMP-2 in the hydrogel layer, long-term osteogenic differentiation of hBMSCs can be sustained, which probably has a key role in the formation of mineralized nodules (Feng et al., 2019). In addition, the crosslinked layer of hydrogels provides suitable biological strength for cell adhesion, infiltration, and proliferation (Zhang et al., 2018). In brief, double-crosslinked coating/HA could promote the greatest amount of osteogenesis, especially the outer hydrogel layer such as Alg70-Gel30 and Alg50-Gel50.

## Conclusion

In this research, we established a bilayer coating with double-crosslinked hydrogels (alginate-GelMA) containing BMP-2 proteins (alginate-GelMA/HA/BMP-2), which displayed great biocompatibility and osteogenesis. The characterization of the coating improved the properties and enhanced the wettability of the native PLLA. To evaluate the biosafety and inductive ability of osteogenesis, the behavior (viability, adherence, and proliferation) and morphology of hBMSCs on the bilayer coatings were tested by multiple exams. The satisfactory function of osteogenesis was verified in bilayer coatings, especially in the specific ratio of alginate to GelMA. We found the best ratios between GelMA and alginate for biological applications. The Alg70-Gel30 and Alg50-Gel50 groups facilitated the osteogenic transformation of hBMSCs. In brief, alginate-GelMA/HA/BMP-2 could increase the hBMSCs’ early transformation of osteoblast lineage and promote the osteogenesis of bone defect, especially the outer hydrogel layer such as Alg70-Gel30 and Alg50-Gel50.

## Data Availability

The original contributions presented in the study are included in the article/Supplementary Material, and further inquiries can be directed to the corresponding authors.

## References

[B1] Ahn S. H. Rath M. Tsao C.-Y. Bentley W. E. Raghavan S. R. (2021). Single-Step Synthesis of Alginate Microgels Enveloped with a Covalent Polymeric Shell: A Simple Way to Protect Encapsulated Cells. ACS Appl. Mater. Inter. 13 (16), 18432–18442. 10.1021/acsami.0c20613 33871957

[B2] Ashwin B. Abinaya B. Prasith T. P. Chandran S. V. Yadav L. R. Vairamani M. (2020). 3D-poly (Lactic Acid) Scaffolds Coated with Gelatin and Mucic Acid for Bone Tissue Engineering. Int. J. Biol. Macromolecules 162, 523–532. 10.1016/j.ijbiomac.2020.06.157 32569692

[B3] Blatchley M. R. Hall F. Ntekoumes D. Cho H. Kailash V. Vazquez‐Duhalt R. (2021). Discretizing Three‐Dimensional Oxygen Gradients to Modulate and Investigate Cellular Processes. Adv. Sci. 8 (14), 2100190. 10.1002/advs.202100190 PMC8292886 34151527

[B4] Cao L. Duan P. G. Wang H. R. Li X. L. Yuan F. L. Fan Z. Y. (2012). Degradation and Osteogenic Potential of a Novel Poly(lactic Acid)/nano-Sized β-tricalcium Phosphate Scaffold. Ijn 7, 5881–5888. 10.2147/IJN.S38127 23226019 PMC3513910

[B5] Chen S. Carlson M. A. Li X. Siddique A. Zhu W. Xie J. (2021). Minimally Invasive Delivery of 3D Shape Recoverable Constructs with Ordered Structures for Tissue Repair. ACS Biomater. Sci. Eng. 7, 2204–2211. 10.1021/acsbiomaterials.1c00344 33929841 PMC8344192

[B6] Chen X. Zhu L. Liu H. Wen W. Li H. Zhou C. (2019). Biomineralization Guided by Polydopamine-Modifed poly(L-Lactide) Fibrous Membrane for Promoted Osteoconductive Activity. Biomed. Mater. 14 (5), 055005. 10.1088/1748-605X/ab2f2d 31271155

[B7] Craciun A.-M. Barhalescu M. L. Agop M. Ochiuz L. Medicine M. M. I. (2019). Theoretical Modeling of Long-Time Drug Release from Nitrosalicyl-Imine-Chitosan Hydrogels through Multifractal Logistic Type Laws. Comput. Math. Methods Med. 2019 (2), 1–10. 10.1155/2019/4091464 PMC6710764 31485257

[B8] Drury J. L. Dennis R. G. Mooney D. J. (2004). The Tensile Properties of Alginate Hydrogels. Biomaterials 25 (16), 3187–3199. 10.1016/j.biomaterials.2003.10.002 14980414

[B9] Feng Q. Xu J. Zhang K. Yao H. Zheng N. Zheng L. (2019). Dynamic and Cell-Infiltratable Hydrogels as Injectable Carrier of Therapeutic Cells and Drugs for Treating Challenging Bone Defects. ACS Cent. Sci. 5 (3), 440–450. 10.1021/acscentsci.8b00764 30937371 PMC6439455

[B10] Golafshan N. Kharaziha M. Fathi M. (2017). Tough and Conductive Hybrid Graphene-PVA: Alginate Fibrous Scaffolds for Engineering Neural Construct. Carbon 111, 752–763. 10.1016/j.carbon.2016.10.042

[B11] Gregurec D. Wang G. Pires R. H. Kosutic M. Lüdtke T. Delcea M. (2016). Bioinspired Titanium Coatings: Self-Assembly of Collagen-Alginate Films for Enhanced Osseointegration. J. Mater. Chem. B 4 (11), 1978–1986. 10.1039/c6tb00204h 32263075

[B12] Hsu C.-S. Haag S. L. Bernards M. T. Li Q. (2020). Evaluation of Chlorine Substituted Hydroxyapatite (ClHAP)/polydopamine Composite Coatings on Ti64. Colloids Surf. B: Biointerfaces 189, 110799. 10.1016/j.colsurfb.2020.110799 32058249

[B13] Hu Y. Cai K. Luo Z. Xu D. Xie D. Huang Y. (2012). TiO2 Nanotubes as Drug Nanoreservoirs for the Regulation of Mobility and Differentiation of Mesenchymal Stem Cells. Acta Biomater. 8 (1), 439–448. 10.1016/j.actbio.2011.10.021 22040682

[B14] Kaito T. Myoui A. Takaoka K. Saito N. Nishikawa M. Tamai N. (2005). Potentiation of the Activity of Bone Morphogenetic Protein-2 in Bone Regeneration by a PLA-PEG/hydroxyapatite Composite. Biomaterials 26 (1), 73–79. 10.1016/j.biomaterials.2004.02.010 15193882

[B15] Kandziora F. Pflugmacher R. Scholz M. Eindorf T. Schnake K. J. Haas N. P. (2004). Bioabsorbable Interbody Cages in a Sheep Cervical Spine Fusion Model. Spine 29 (17), 1845–1855. discussion 1856. 10.1097/01.brs.0000137060.79732.78 15534403

[B16] Kraehenbuehl T. P. Zammaretti P. Van der Vlies A. J. Schoenmakers R. G. Lutolf M. P. Jaconi M. E. (2008). Three-dimensional Extracellular Matrix-Directed Cardioprogenitor Differentiation: Systematic Modulation of a Synthetic Cell-Responsive PEG-Hydrogel. Biomaterials 29 (18), 2757–2766. 10.1016/j.biomaterials.2008.03.016 18396331

[B17] Lee H. Dellatore S. M. Miller W. M. Messersmith P. B. (2007). Mussel-inspired Surface Chemistry for Multifunctional Coatings. Science 318 (5849), 426–430. 10.1126/science.1147241 17947576 PMC2601629

[B18] Li M. Liu X. Xu Z. Yeung K. W. K. Wu S. (2016). Dopamine Modified Organic-Inorganic Hybrid Coating for Antimicrobial and Osteogenesis. ACS Appl. Mater. Inter. 8 (49), 33972–33981. 10.1021/acsami.6b09457 27960367

[B19] Liang Y. Chen B. Li M. He J. Yin Z. Guo B. (2020). Injectable Antimicrobial Conductive Hydrogels for Wound Disinfection and Infectious Wound Healing. Biomacromolecules 21 (5), 1841–1852. 10.1021/acs.biomac.9b01732 32388998

[B20] Lin H.-H. Hsieh F.-Y. Tseng C.-S. Hsu S.-h. (2016). Preparation and Characterization of a Biodegradable Polyurethane Hydrogel and the Hybrid Gel with Soy Protein for 3D Cell-Laden Bioprinting. J. Mater. Chem. B 4 (41), 6694–6705. 10.1039/c6tb01501h 32263524

[B21] Nair M. S. Tomar M. Punia S. Kukula-Koch W. Kumar M. (2020). Enhancing the Functionality of Chitosan- and Alginate-Based Active Edible Coatings/films for the Preservation of Fruits and Vegetables: A Review. Int. J. Biol. Macromolecules 164, 304–320. 10.1016/j.ijbiomac.2020.07.083 32682968

[B22] Nichol J. W. Koshy S. T. Bae H. Hwang C. M. Yamanlar S. Khademhosseini A. (2010). Cell-laden Microengineered Gelatin Methacrylate Hydrogels. Biomaterials 31 (21), 5536–5544. 10.1016/j.biomaterials.2010.03.064 20417964 PMC2878615

[B23] Niu X. Feng Q. Wang M. Guo X. Zheng Q. (2009). Porous Nano-HA/collagen/PLLA Scaffold Containing Chitosan Microspheres for Controlled Delivery of Synthetic Peptide Derived from BMP-2. J. Controlled Release 134 (2), 111–117. 10.1016/j.jconrel.2008.11.020 19100794

[B24] Oliveira F. C. Carvalho J. O. Magalhães L. S. S. M. da Silva J. M. Pereira S. R. Gomes Júnior A. L. (2021). Biomineralization Inspired Engineering of Nanobiomaterials Promoting Bone Repair. Mater. Sci. Eng. C 120, 111776. 10.1016/j.msec.2020.111776 33545906

[B25] Pacelli S. Basu S. Berkland C. Wang J. Paul A. (2018). Design of a Cytocompatible Hydrogel Coating to Modulate Properties of Ceramic-Based Scaffolds for Bone Repair. Cel. Mol. Bioeng. 11 (3), 211–217. 10.1007/s12195-018-0521-3 PMC6188648 30338007

[B26] Piluso S. Skvortsov G. A. Altunbek M. Afghah F. Khani N. Koç B. (2021). 3D Bioprinting of Molecularly Engineered PEG-Based Hydrogels Utilizing Gelatin Fragments. Biofabrication 13 (4), 045008. 10.1088/1758-5090/ac0ff0 34192670

[B27] Rahali K. Ben Messaoud G. Kahn C. Sanchez-Gonzalez L. Kaci M. Cleymand F. (2017). Arab-tehrany, E. (Synthesis and Characterization of Nanofunctionalized Gelatin Methacrylate Hydrogels. Ijms 18 (12), 2675. 10.3390/ijms18122675 PMC5751277 29232870

[B28] Rai A. Pinto S. Evangelista M. B. Gil H. Kallip S. Ferreira M. G. S. (2016). High-density Antimicrobial Peptide Coating with Broad Activity and Low Cytotoxicity against Human Cells. Acta Biomater. 33, 64–77. 10.1016/j.actbio.2016.01.035 26821340

[B29] Silva J. M. García J. R. Reis R. L. García A. J. Mano J. F. (2017). Tuning Cell Adhesive Properties via Layer-By-Layer Assembly of Chitosan and Alginate. Acta Biomater. 51, 279–293. 10.1016/j.actbio.2017.01.058 28126597 PMC5665021

[B30] Simó G. Fernández‐Fernández E. Vila‐Crespo J. Ruipérez V. Rodríguez‐Nogales J. M. (2017). Research Progress in Coating Techniques of Alginate Gel Polymer for Cell Encapsulation. Carbohydr. Polym. 170, 1–14. 10.1016/j.carbpol.2017.04.013 28521974

[B31] Sobolev A. Valkov A. Kossenko A. Wolicki I. Zinigrad M. Borodianskiy K. (2019). Bioactive Coating on Ti Alloy with High Osseointegration and Antibacterial Ag Nanoparticles. ACS Appl. Mater. Inter. 11 (43), 39534–39544. 10.1021/acsami.9b13849 31590486

[B32] Szustakiewicz K. Włodarczyk M. Gazińska M. Rudnicka K. Płociński P. Szymczyk-Ziółkowska P. (2021). The Effect of Pore Size Distribution and L-Lysine Modified Apatite Whiskers (HAP) on Osteoblasts Response in PLLA/HAP Foam Scaffolds Obtained in the Thermally Induced Phase Separation Process. Ijms 22 (7), 3607. 10.3390/ijms22073607 33808501 PMC8036975

[B33] Tan W. Gao C. Feng P. Liu Q. Liu C. Wang Z. (2021). Dual-functional Scaffolds of poly(L-Lactic Acid)/nanohydroxyapatite Encapsulated with Metformin: Simultaneous Enhancement of Bone Repair and Bone Tumor Inhibition. Mater. Sci. Eng. C 120, 111592. 10.1016/j.msec.2020.111592 33545810

[B34] Tas A. C. Bhaduri S. B. (2004). Rapid Coating of Ti6Al4V at Room Temperature with a Calcium Phosphate Solution Similar to 10× Simulated Body Fluid. J. Mater. Res. 19 (9), 2742–2749. 10.1557/JMR.2004.0349

[B35] Tsai W.-B. Chien C.-Y. Thissen H. Lai J.-Y. (2011). Dopamine-assisted Immobilization of Poly(ethylene Imine) Based Polymers for Control of Cell-Surface Interactions. Acta Biomater. 7 (6), 2518–2525. 10.1016/j.actbio.2011.03.010 21402183

[B36] Tsai Y.-C. Li S. Hu S.-G. Chang W.-C. Jeng U.-S. Hsu S.-h. (2015). Synthesis of Thermoresponsive Amphiphilic Polyurethane Gel as a New Cell Printing Material Near Body Temperature. ACS Appl. Mater. Inter. 7 (50), 27613–27623. 10.1021/acsami.5b10697 26651013

[B37] Wang X. Guan S. Zhang K. Li J. (2020). Benlysta-Loaded Sodium Alginate Hydrogel and its Selective Functions in Promoting Skin Cell Growth and Inhibiting Inflammation. ACS Omega 5 (18), 10395–10400. 10.1021/acsomega.0c00283 32426596 PMC7226882

[B38] Wang Z. Chen L. Wang Y. Chen X. Zhang P. (2016). Improved Cell Adhesion and Osteogenesis of Op-HA/PLGA Composite by Poly(dopamine)-Assisted Immobilization of Collagen Mimetic Peptide and Osteogenic Growth Peptide. ACS Appl. Mater. Inter. 8 (40), 26559–26569. 10.1021/acsami.6b08733 27649958

[B39] Wu Y. Zhang Y. Zhang R. Chen S. (2021). Preparation and Properties of Antibacterial Polydopamine and Nano-Hydroxyapatite Modified Polyethylene Terephthalate Artificial Ligament. Front. Bioeng. Biotechnol. 9, 630745. 10.3389/fbioe.2021.630745 33869151 PMC8044552

[B40] Xing H. Li R. Wei Y. Ying B. Li D. Qin Y. (2020). Improved Osteogenesis of Selective-Laser-Melted Titanium Alloy by Coating Strontium-Doped Phosphate with High-Efficiency Air-Plasma Treatment. Front. Bioeng. Biotechnol. 8 (367). 10.3389/fbioe.2020.00367 PMC7235326 32478042

[B41] Zhang J. Zhang W. Bao T. Chen Z. (2014). Mussel-inspired Polydopamine-Assisted Hydroxyapatite as the Stationary Phase for Capillary Electrochromatography. Analyst 139 (1), 242–250. 10.1039/c3an01668d 24195104

[B42] Zhang K. Jia Z. Yang B. Feng Q. Xu X. Yuan W. (2018). Adaptable Hydrogels Mediate Cofactor-Assisted Activation of Biomarker-Responsive Drug Delivery via Positive Feedback for Enhanced Tissue Regeneration. Adv. Sci. 5 (12), 1800875. 10.1002/advs.201800875 PMC6299823 30581701

[B43] Zhang Y. Li R. Wu W. Qing Y. a. Tang X. Ye W. (2018). Adhesion and Proliferation of Osteoblast-like Cells on Porous Polyetherimide Scaffolds. Biomed. Res. Int. 2018, 1–7. 10.1155/2018/1491028 PMC6288576 30598988

